# ^125^I brachytherapy: a useful treatment to control painful osteoblastic bone metastases

**DOI:** 10.1007/s12094-022-03025-0

**Published:** 2022-12-06

**Authors:** Yiming Liu, Chengzhi Zhang, Kaihao Xu, Kunpeng Wu, Xinwei Han, Dechao Jiao

**Affiliations:** The Department of Interventional Radiology, The First Affiliated Hospital of Zhengzhou University, 1 Jianshe East Road, Zhengzhou, 450052 China

**Keywords:** Osteoblastic bone metastases, Relief of pain, ^125^I brachytherapy, Cone beam CT, Logistic regression analysis

## Abstract

**Backgrounds:**

^125^I brachytherapy is effective in relieving cancer pain due to osteolytic bone metastases. However, fewer studies focused on painful osteoblastic bone metastases (OBMs), we conducted a retrospective study to evaluate the efficacy of ^125^I brachytherapy for the treatment of painful OBMs.

**Methods:**

From April 2017 to April 2019, clinical data of a total of 65 patients with OBMs who underwent CT/cone beam CT -guided ^125^I brachytherapy were collected and analyzed. The primary study endpoints were technical success, relief of pain (RoP), and quality of life (QoL). The secondary study endpoints were treatment-related complications, local tumor control (LCR), and overall survival (OS). The logistic regression analysis was performed to predict RoP.

**Results:**

Technical success rate was 100%. Visual analog scale scores and daily morphine consumption continuously decreased significantly at 2 weeks, 6 weeks, and 10 weeks (all *P* < 0.05). The RoP at 6 weeks was 84.62%. QoL presented improvement at 6 and 10 weeks. Only minor complications occurred in 12 patients (18.46%). LCR was 93.85% at 10 weeks. The OS was 29.80 months. Two factors were significantly associated with the RoP: max diameter (MD, < 3 cm vs. ≥ 3 cm, *P* = 0.019) and serum levels of bone alkaline phosphatase (B-ALP, ≥ 100 U/L vs. < 100 U/L, *P* = 0.016).

**Conclusions:**

^125^I brachytherapy is an effective treatment in relieving painful OBMs and improving patients’ QoL.

## Introduction

About 75% of patients with advanced malignant tumors will suffer from moderate to severe tumor-related bone pain, and bone metastasis occurred is one of the most common causes [1]. Osteoblastic bone metastases (OBMs), which account for approximately 20–30% of bone metastases [2], primarily originate from prostate cancer, breast cancer, and lung cancer [3]. Since patients with osteoblastic bone metastases are less likely to die in the short term, relief of pain (RoP) becomes the primary treatment goal for this patients. Although External Beam Radiation Therapy (EBRT) is a standard and effective local treatment for pain palliation in OBMs, it only showed a 17% complete response rate and a 66% overall response rate at 3 months in a prospective, three-stage randomized controlled study by Hartsell et al. [4]. Therefore, for those who cannot undergo surgical resection and EBRT, or EBRT failure, cancerous bone pain becomes the major difficulty encountered by patients and physicians, and it will severely affect the patient's quality of life (QoL).

Brachytherapy has been confirmed to be an effective alternative therapy for RoP and has been used to treat cancer pain caused by pancreatic cancer, retroperitoneal lymph node metastases, and bone metastases [5]. ^125^I seeds, a brachytherapy nuclide with low energy and a moderate half-life (59.6 days), can continuously emit a low dose of γ-rays and gradually accumulate in the tumor tissue. Thus, it not only kills tumor cells and reduces their compression on surrounding nerves or organs, but also inhibits the release of pain-causing factors secreted by tumor cells [6]. Shi et al. reported that computer tomography (CT)-guided ^125^I brachytherapy for EBRT-refractory bone metastases resulted in a 73% reduction in brief pain inventory (BPI) after 24 weeks compared to pre-treatment (*P* = 0.001) [7]. In a controlled study [8], VAS (visual analog scale) was significantly lower in patients in both the ^125^I seeds treatment and EBRT groups, but the proportion of patients with VAS for worst pain was lower in the former group: 27.3% vs. 30.6% at 4 weeks (*P* < 0.01), 12.2% vs. 26.0% at 6 weeks (*P* < 0.01), 11.4% vs. 23.3% at 8 weeks (*P* < 0.01), demonstrating significant pain relief with ^125^I brachytherapy. Another advantage of brachytherapy is local, continuous, and low-dose radiation exposure to avoid off-target effects that can damage surrounding normal tissues, which is especially important for bone metastases that occur mostly in the vertebrae and pelvis. Moreover, ^125^I brachytherapy allows for an excellent local control rate (LCR) of tumors. In a retrospective study of ^125^I brachytherapy for spinal metastases reported by Liu et al. [9], LCR reached 91.3%, 81.9%, and 81.9% at 3 months, 6 months, and 12 months, respectively.

However, in previous studies related to ^125^I brachytherapy for bone metastases, most lesions showed osteolytic changes, and studies of osteoblastic bone metastases are rare. In addition, clinical outcomes may be influenced by pre-treatment characteristics at the time of diagnosis as well as technical parameters of ^125^I brachytherapy. Therefore, this study aims to evaluate the RoP efficacy of ^125^I brachytherapy in patients with unresectable or EBRT-refractory OBMs and to identify the prognostic factors for RoP after ^125^I brachytherapy.

## Methods

### Patients

**Fig. 1:**
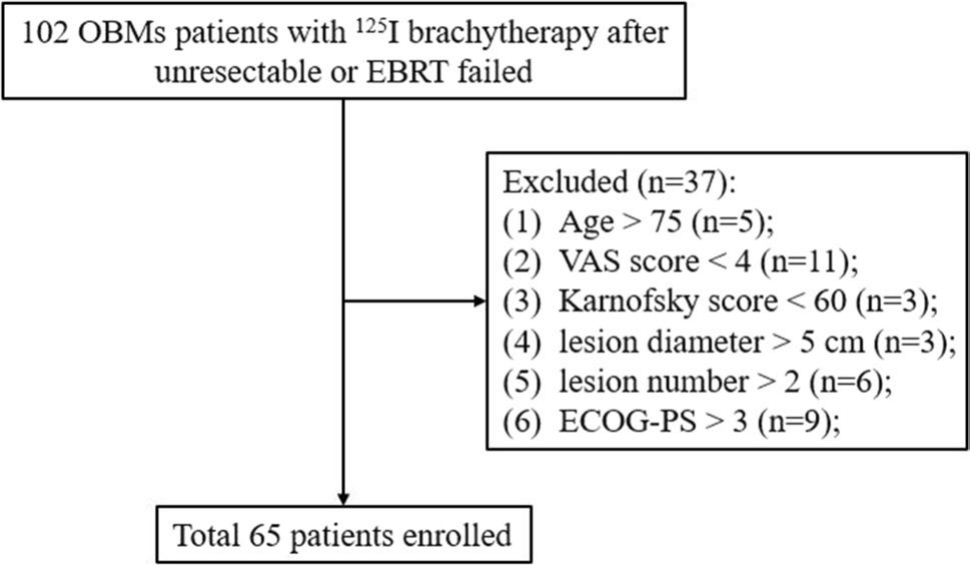
The flow chart of this study is shown

Clinical data were collected from OBMs patients with unresectable or EBRT failed who underwent CT/cone beam CT (CBCT)-guided ^125^I brachytherapy at our center from April 1, 2017 to April 1, 2019. The workflow diagram is illustrated in Fig. 1. The inclusion criteria are as follows: (1) age of 15–75 years old; (2) imaging/pathology/medical history suggesting malignant bone metastasis; (3) VAS score ≥ 4; (4) pain is associated with the bone metastases; (5) Karnofsky score ≥ 60; (6) Eastern Cooperative Oncology Group performance status (ECOG-PS) score ≤ 3; and (7) lesion diameter ≤ 5 cm and lesion number ≤ 2. The exclusion criteria were as follows: (1) severe coagulation dysfunction (prothrombin time ≥ 21 s, PLT < 30 × 10^9^/L); (2) severe cardiopulmonary dysfunction; and (3) presence of impending fracture. This retrospective study was approved by the Clinical Ethics Committees of the First Affiliated Hospital of Zhengzhou University (Ethical review number: 2016-KY-212).

### ^125^I seeds

^125^I seeds (Tianjin Saide Biopharmaceutical Co., Ltd., China) were Type-6711 and cylindrical (0.8 mm × 4.5 mm) with titanium capsules. The radioactivity per seed ranged from 0.75 to 0.80 mCi with a half-life of 59.6 days. They emit low-dose γ-rays (35.5 keV) and soft X-rays (28.6 keV). The radius of the effective antitumor activity was 17 mm, and more than 90% of the brachytherapy dose energy was released within 10 months.

### Preoperative preparation

Before the ^125^I seeds implantation (ISI), each patient underwent CT (16-row Brilliance CT, Netherlands)/CBCT [an auxiliary function of the digital angiography machine Artis zeego with iGuide virtual navigation (Siemens, Germany)] to evaluate the detailed location and volume of the tumors. Then, the pre-treatment plan and postoperative dose verification were performed using a computerized treatment plan system (TPS, Beijing Atom & High Technical Industries Inc., China). The clinical target volume (CTV) was defined as a 1.0 cm expansion external to the gross tumor volume (GTV). The planned target volume (PTV) was defined as a 0.5 cm expansion external to the CTV. According to our previous experience, the prescribed dose was set at 120 Gy and considering their tolerability, the prescribed dose was set at 70–90 Gy for patients after EBRT failure. In addition, the matched peripheral dose was set at 110–140 Gy to ensure a sufficient 90% volume of the tumor-absorbed dose (D90). Organs at risk (OAR) are defined as important or irradiation-sensitive organs around osteoblastic bone metastases, such as the spinal cord, nerve, heart, and blood vessels.

### ISI procedure

**Fig. 2:**
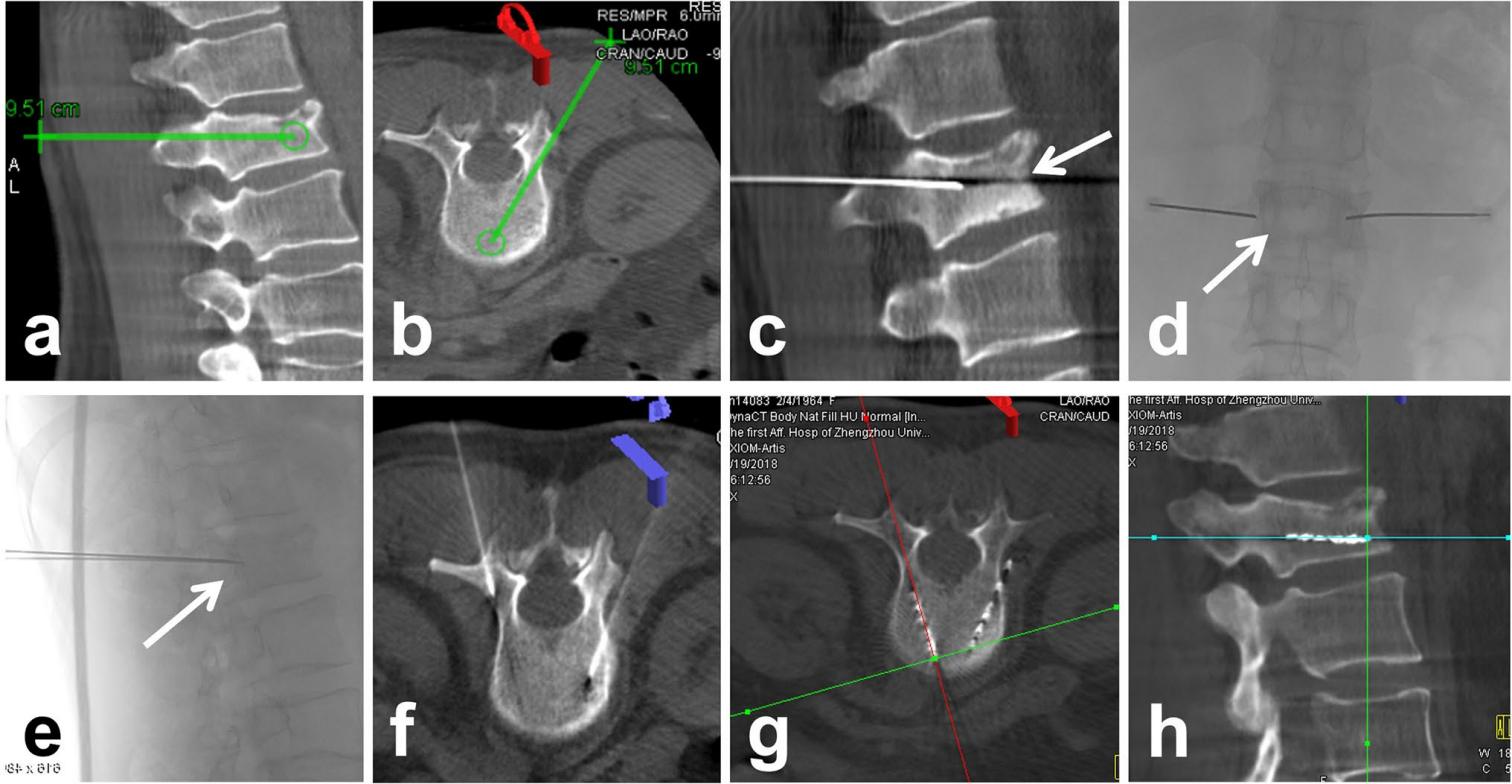
Sagittal (**a**) and axial (**b**) iGuide images showing the location of the lesion and the simulated puncture path in a 66-year-old patient with lumbar metastases from prostate cancer. Based on the TPS, sagittal CBCT images (**c**) as well as coronal (**d**) and sagittal (**e**) digital subtraction images of the puncture procedure (white arrow) were illustrated. The axial CBCT image (**f**) showed the second puncture needle entry. Axial (**g**) and sagittal (**h**) CBCT images of the seeds chain implantation

**Fig. 3:**
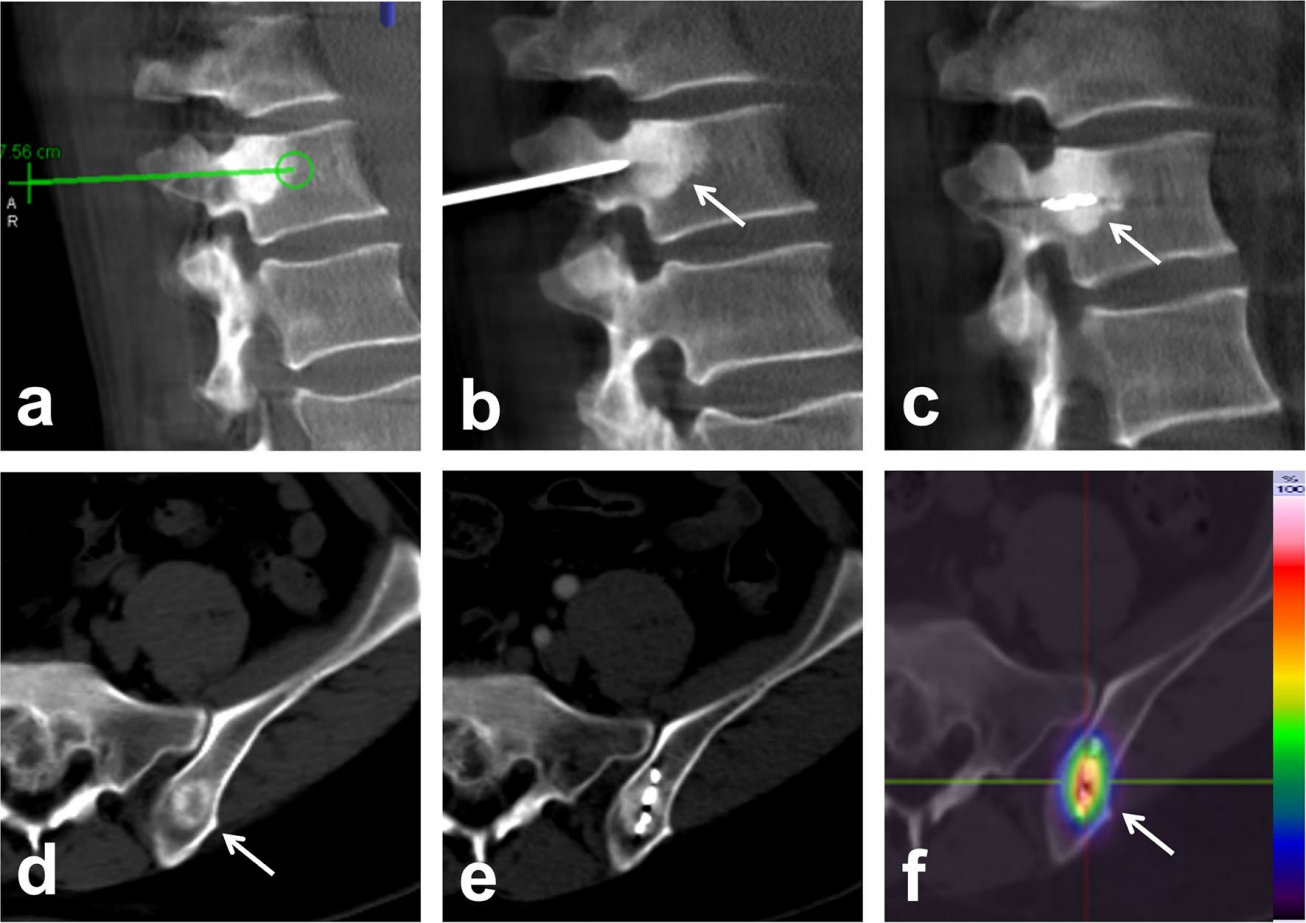
A 63-year-old female patient with lumbar spine and iliac bone metastases from breast cancer. The CT images (**a**–**c**) showed the location of the metastatic tumor foci in the lumbar spine, the virtual puncture path, and guidance of  the puncture and ISI. CT images (**d**–**e**) showed the metastatic tumor in the iliac bone and the implanted seeds. Image (**f**) showed the radioactive distribution of the seeds chain after ISI

**Fig. 4:**
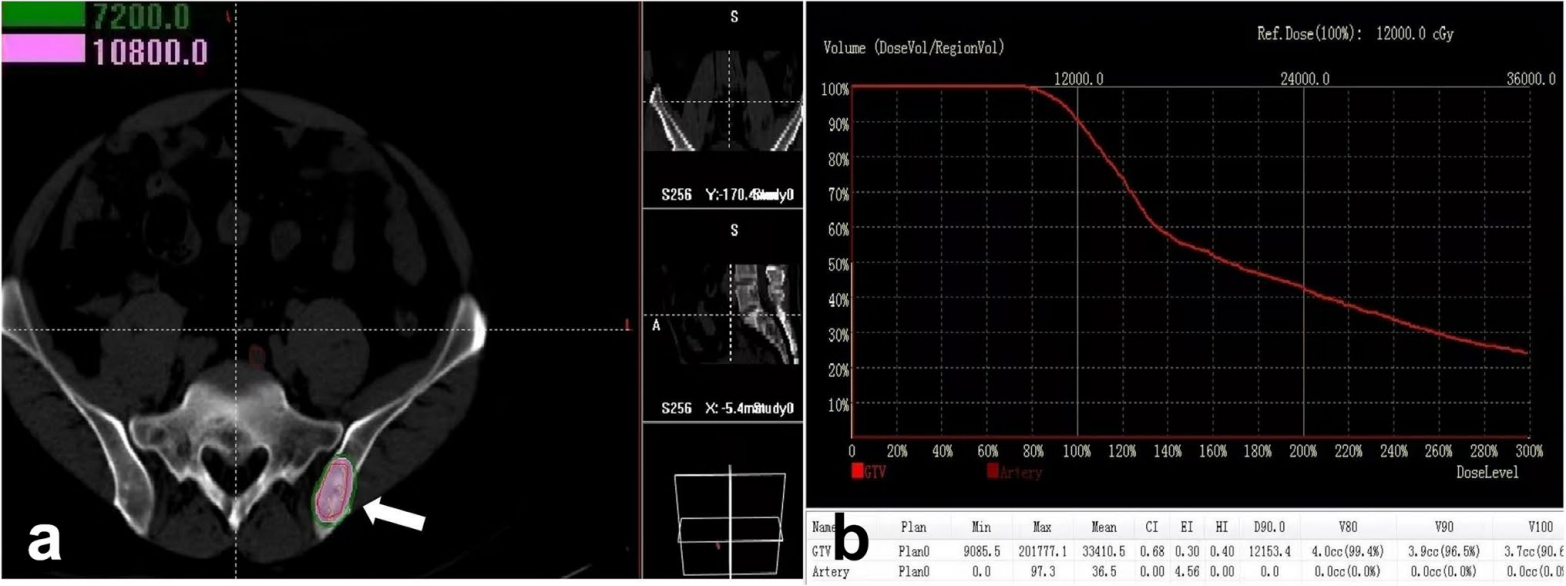
The actual absorbed dose (**a**) and dose–volume histogram (**b**) were verified by the postoperative TPS plan

Two doctors with more than 10 years of experience performed all the operation procedures. The appropriate body position was selected according to the location of the lesions. First, the patient underwent CT/CBCT scanning of the lesions, and then the puncture path was selected according to the preoperative TPS. Second, after local anesthesia with 2% lidocaine (5 ml), based on our clinical experience, an ISI needle (Nanjing Medical Technology Co. Ltd., China) was connected to a medical electric bone saw drill (Hangzhou Yifan Medical Devices Co., China) rotating clockwise or counterclockwise with a small advance for puncture tunnel. Third, the ISI needle was inserted into the farthest end of the lesion, and multiple needles were used at equal intervals in parallel when the transverse diameter of the tumor was larger than 1.5 cm. Fourth, the ISI gun (Tianjin Saide Biopharmaceutical Co. Ltd. China) was attached to the ISI needle, and the seeds were released one by one from the deeper to the shallower layers, with a seed interval distance of 0.5 mm while retracting the ISI needle. Finally, CT/CBCT was performed again to confirm the ^125^I seeds distribution, and dose absorption was performed again on TPS to calculate the D90 and OAR doses (Figs. 2, 3, 4).

### Postoperative evaluation and definition

Dynamic enhanced CT or MRI was performed every month for the first 3 months and every 2 months thereafter. The last follow-up was performed in December 2021. The primary endpoints were technical success rate, RoP, and QoL score. Technical success was defined as the successful completion of ISI. The bone pain was evaluated by the daily morphine consumption (DMC) according to the conversion standard of the NCCN guidelines for adult cancer pain management in 2019 [10] and VAS (0–3 points, mild pain; 4–6 points, moderate pain; 7–10 points, severe pain), which recorded at T0 (pre-treatment), T2 (2 weeks), T6 (6 weeks), and T10 (10 weeks) for each patient. RoP was defined as the reduction in VAS score by more than 2 at T6. The QoL assessment had 5 aspects: sleep, appetite, fatigue, and Karnofsky score. The first 4 indicators were graded using a five-point scale (5 = very good, 4 = normal, 3 = mild, 2 = bad, and 1 = worst) and the Karnofsky score was on a scale of 10 (range: 10–100), which were all recorded at T0, T2, T6, and T10.

The secondary points included treatment-related complications, tumor response, and overall survival (OS). According to the modified Response Evaluation Criteria in Solid Tumors (mRECIST) standard [11], tumor response was assessed by imaging on enhanced CT or MRI (whether new high density on CT or new low signal on MRI emerging) at T10, including complete response (CR), partial response (PR), stable disease (SD), progressive disease (PD), and LCR = [(CR + PR + SD)/total number of lesions] × 100%. Only local progression of lesions with ^125^I brachytherapy was assessed, except for systemic or regional metastases. OS was defined as the time from the date of study entry to the date of death or last follow-up visit. Treatment-related complications were evaluated according to the classification standard of the European Society of Cardiovascular and Interventional Radiology (CIRSE) [12].

### Statistical analysis

Descriptive statistics were used to characterize our population. *Paired t-test* was performed to analyze the changes of DMC at different times. *Wilcoxon test* was used to compare the VAS scores and QoL scores at different times. A *logistic regression analysis* was used to detect the association between treatment characteristics and the RoP. *Kaplan–Meier analysis* (*Log-rank test*) was used to analyze the OS. The covariates that may be associated with outcomes in univariate analyses were included in multivariate models. When 2 metastases were treated in one patient, a single response was recorded for the most painful target area in all clinical evaluation indicators as well as in the statistical models. *P* < 0.05 was considered as statistically significant. The SPSS 24.0 software package (IBM Corp, New York, USA) was used to perform the statistical analysis.

## Results

### General characteristics

The records of 102 OBMs patients with unresectable or EBRT failed who underwent CT/ CBCT-guided ^125^I brachytherapy were retrospectively reviewed. Ultimately, a total of 65 patients were enrolled in this study, and their basic clinical characteristics are shown in Table 1. Of these, 27 patients (41.54%) undergone surgery, 39 patients (60.00%) received EBRT, and all patients (100%) were taking analgesics, but all patients had suboptimal pain control (VAS ≥ 4) and ultimately chose ^125^I brachytherapy. The technical success rate was 100%. By the postoperative TPS system, the mean D90 was (110.9 ± 8.0) Gy (86.5–132.4 Gy), and the mean OAR dose was (4.2 ± 1.0) Gy (1.8–6.1 Gy). The total number of seeds was 1817 with a mean of (19.33 ± 6.51) seeds per lesion.

**Table 1:** Demographics and clinical characteristics

Data	Value, *n* (%)
Age, median, range	60 (42–75)
Sex (Male, Female)	37 (56.92%), 28 (43.08%)
Primary tumor diagnosis	
Breast cancer	26 (40.00%)
Prostate cancer	18 (27.69%)
Lung carcinoma	8 (12.31%)
Thyroid Cancer	7 (10.77%)
Melanoma	6 (9.23%)
Previous treatment (R, EBRT, C, A)	27 (41.54%), 39 (60.00%), 20 (30.77%), 65 (100%)
Metastasis site (Vertebra, Pelvic, Femur, Ribs)	30 (46.15%), 28 (43.08%), 4 (6.15%), 3 (4.62%)
Total number of metastases	94 (100%)
Metastases number (1 lesion, 2 lesions)	36 (55.38%), 29 (44.62%)
Mean max diameter (cm), mean ± SD, range	2.76 ± 0.62, 1.50–4.50
Metastases diameter (< 3.0 cm, ≥ 3.0 cm)	39 (60.00%), 26 (40.00%)
ECOG-PS (0/1/2/3)	18 (27.69%), 21 (32.31%), 15 (23.08%), 11 (16.92%)
B-ALP (< 100 U/L, ≥ 100 U/L)	24 (36.92%), 41 (63.08%)
TP (≥ 66 g/L, < 66 g/L)	26 (40.00%), 39 (60.00%)
ALB (≥ 40 g/L, < 40 g/L)	24 (36.92%), 41 (63.08%)
D90 (≥ 110 Gy, < 110 Gy)	43 (66.15%), 22 (33.85%)
OAR dose (< 4.5 Gy, ≥ 4.5 Gy)	42 (64.62%), 23 (35.38%)

*SD* standard deviation, *R* Resection, *EBRT* External beam radiation therapy, *C* Chemotherapy, *A* Analgesic drugs, *ECOG-PS* Eastern Cooperative Oncology Group performance status, *B-ALP* Serum levels of bone alkaline phosphatase, *TP* serum total protein concentration, *ALB* serum albumin concentration, *D90* 90% volume of the tumor-absorbed dose, *OAR* organs at risk

### RoP and associated factors

**Fig. 5:**
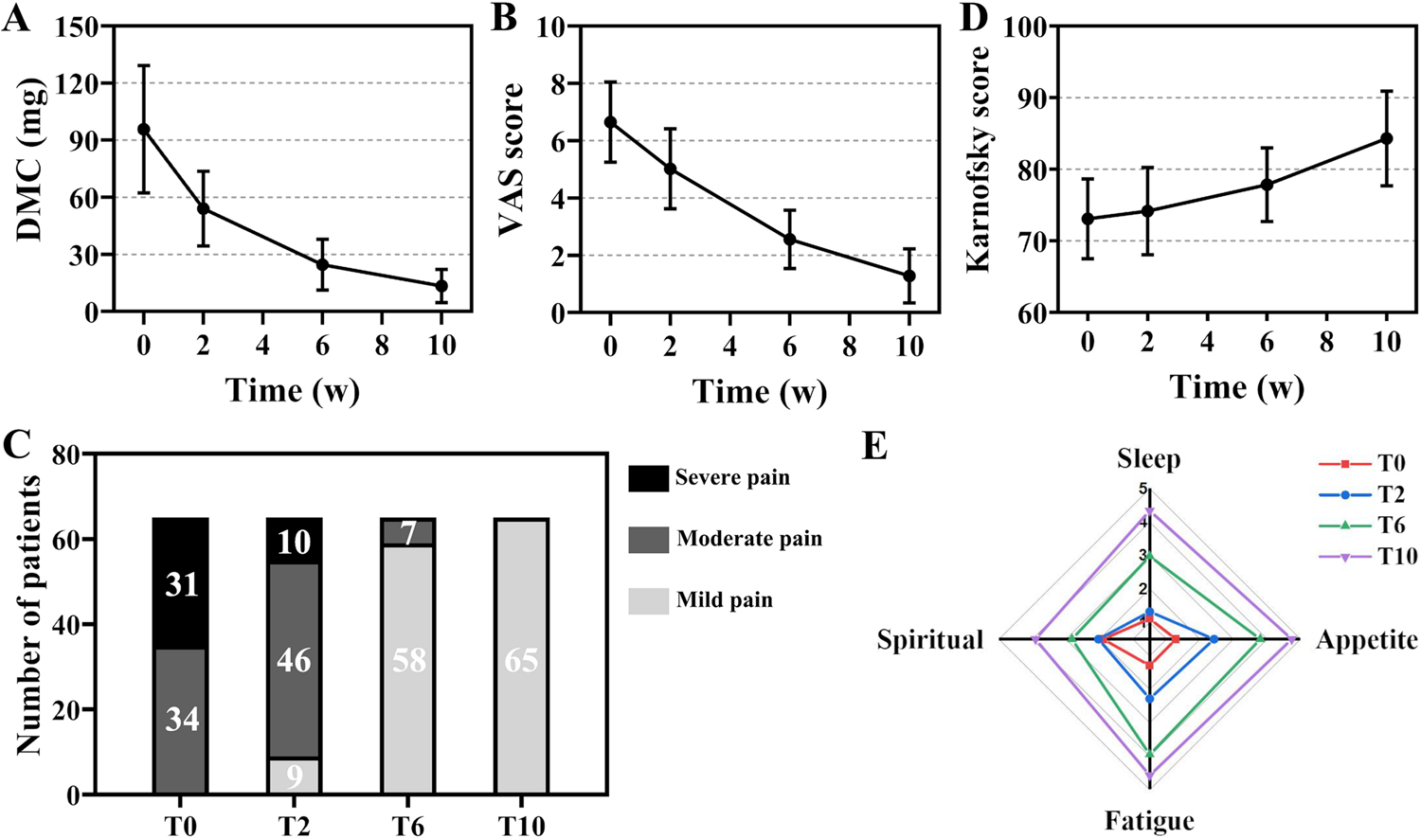
The changes in the indicators at different times. The trends in daily morphine consumption (DMC, **a**) and visual analog scale (VAS, **b**) scores of all patients at T0, T2, T6, and T10 (all *P* = 0.00). The proportion of different levels of pain in VAS scores at T0, T2, T6, and T10 (all *P* = 0.00, **c**). The trends in Karnofsky score of all patients at T0, T2 (*P* = 0.18), T6 (*P* = 0.00), and T10 (*P* = 0.00) (**d**). The trends at T0, T2, T6, and T10 in the other four dimensions of QoL scores: sleep, appetite, fatigue, and spiritual (radar diagram, all *P* = 0.00, **e**)

The validity of pain relief is summarized in Table 2. From (95.69 ± 33.45) mg at T0, DMC was gradually reduced at T2 (*P* = 0.00) and T6 (*P* = 0.00) and was as low as (13.38 ± 8.71) mg at T10 (*P* = 0.00), without rebound (Fig. 5A). Meanwhile, as the DMC dose was reduced, the analgesic-related adverse events were correspondingly reduced (Table 3).

**Table 2:** Bone pain evaluation at different times

Indicators, mean ± SD	T0	T2 *t*/*Z* value, *P-*value	T6 *t*/*Z* value, *P-*value	T10 *t*/*Z* value, *P-*value
DMC (mg)	95.69 ± 33.45	54.00 ± 19.67, *t* = 13.14, *P* = 0.00^***a***^	24.62 ± 13.36, *t* = 16.03, *P* = 0.00^***b***^	13.38 ± 8.71, *t* = 18.82, *P* = 0.00^***c***^
VAS score	6.65 ± 1.40	5.02 ± 1.40, *Z* = − 7.23*, P* = 0.00^***a***^	2.55 ± 1.02, *Z* = − 7.20, *P* = 0.00^***b***^	1.28 ± 0.94, *Z* = −7.04, *P* = 0.00^***c***^
Mild pain (0–3)	0	2.78 ± 0.44	2.34 ± 0.76	1.28 ± 0.94
Moderate pain (4–6)	5.60 ± 0.65	4.98 ± 0.80	4.67 ± 0.82	0
Severe pain (7–10)	7.87 ± 0.97	7.20 ± 0.42	0	0

^a^VAS score at T2 compared with that at T0; ^b^VAS score at T6 compared with that at T0; ^c^VAS score at T10 compared with that at T0. *SD* standard deviation, *DMC* daily morphine consumption, *VAS* visual analog scale

**Table 3:** Analgesic-related adverse events at different times

Adverse events	T0	T2	T6	T10
Nausea	24 (36.92%)	16 (24.62%)	10 (15.38%)	8 (12.31%)
Vomiting	21 (32.31%)	17 (26.15%)	11 (16.92%)	5 (7.69%)
Constipation	18 (27.69%)	13 (20.00%)	6 (9.23%)	5 (7.69%)
Fatigue	10 (15.38%)	10 (15.38%)	0	0
Dizzy	12 (18.46%)	8 (12.31%)	0	0

In addition, compared to T0, VAS were significantly decreased at T2 (*P* = 0.00), T6 (*P* = 0.00), and T10 (*P* = 0.00), detailed in Fig. 5B. Among them, the number of patients with severe pain decreased from 31 (47.69%) at T0 to 10 (15.38%) at T2 (*P* = 0.00), and no more patients with severe pain at T6 (*P* = 0.00). At T6, the RoP rate was 84.62%, and all patients performed mild pain at T10 (Fig. 5C). Univariate analysis (Table 4) showed that max diameter (MD) and serum levels of bone alkaline phosphatase (B-ALP) were significant predictive factors for RoP (*P* < 0.05). A further multivariate logistic regression analysis exhibited that MD < 3 cm (OR 9.541, 95%CI 1.459–62.380) and serum levels of bone alkaline phosphatase (B-ALP) ≥ 100 U/L (OR 0.102, 95%CI 0.016–0.657) were associated with better relief of pain (*P* = 0.019 and *P* = 0.016, respectively).

**Table 4:** Univariate and multivariate logistic regression on RoP

Factors	Univariate		Multivariate	
	OR (95% CI)	*P*-value	OR (95% CI)	*P*-value
Gender (male, female)	1.161 (0.294–4.583)	0.831	–	–
Age (< 60 years, ≥ 60 years)	1.490 (0.372–5.973)	0.573	–	–
ECOG-PS (< 2, ≥ 2)	2.429 (0.613–9.626)	0.207	–	–
Tumor metastasis site	–	–	–	–
Vertebra	1.937 (0.491–7.646)	0.345	–	–
Pelvic bones	2.250 (0.569–8.902)	0.248	–	–
Max diameter (< 3.0 cm, ≥ 3.0 cm)	**4.421 (1.024–19.080)**	**0.046**	**9.541 (1.459–62.380)**	**0.019**
Number of metastases (1, 2)	0.478 (0.112–2.043)	0.319	–	–
B-ALP (< 100 U/L, ≥ 100 U/L)	**0.192 (0.044–0.833)**	**0.027**	**0.102 (0.016–0.657)**	**0.016**
TP (≥ 66 g/L, < 66 g/L)	1.619 (0.418–6.268)	0.485	–	–
ALB (< 40 g/L, ≥ 40 g/L)	3.083 (0.772–12.316)	0.111	–	–
D90 (≥ 110 Gy, < 110 Gy)	0.438 (0.085–2.264)	0.324	–	–
OAR dose (< 4.5 Gy, ≥ 4.5 Gy)	0.167 (0.020–1.410)	0.100	–	–

*OR* odds ratio, *CI* confidence interval, *ECOG-PS* Eastern Cooperative Oncology Group Performance Status, *B-ALP* serum levels of bone alkaline phosphatase, *TP* serum total protein concentration, *ALB* serum albumin concentration, *D90* 90% volume of the tumor-absorbed dose, *OAR* organs at risk

### QoL scores and treatment-related complications

A comparison of QoL scores at different times (Table 5) revealed that rapid recovery of sleep, appetite, fatigue, and spirituality were all significant at T2, T6, and T10, with all comparisons statistically different from the scores at T0 (all *P* = 0.00). However, there was no significant difference in the Karnofsky scores between T0 and T2 (*P* = 0.18), while the scores were elevated at T6 and T10 (all *P* = 0.00) (Fig. 5D–E). Minor subcutaneous hemorrhage occurred in 7 patients (10.77%), 3 patients (4.62%) had mild fever and 2 patients (3.08%) had local skin reactions, which were all cured with symptomatic treatment. No serious treatment-related complications such as spinal cord injury, hemorrhage, or radiological damage to vital organs occurred.

**Table 5:** QoL evaluation at different times

Indicators, mean ± SD, *t*/*Z* value, *P-*value	T0	T2	T6	T10
QoL scores				
Sleep	1.11 ± 0.31	1.32 ± 0.47, *Z* = – 3.30, *P* = 0.00^a^	2.98 ± 0.60, *Z* = – 7.17, *P* = 0.00^b^	4.34 ± 0.59, *Z* = – 7.25, *P* = 0.00^c^
Appetite	1.28 ± 0.55	2.42 ± 0.58, *Z* = – 7.44, *P* = 0.00^a^	3.80 ± 0.56, *Z* = – 7.13, *P* = 0.00^b^	4.74 ± 0.54, *Z* = – 7.22, *P* = 0.00^c^
Fatigue	1.29 ± 0.52	2.28 ± 0.52, *Z* = – 7.41, *P* = 0.00^a^	3.95 ± 0.69, *Z* = – 7.16, *P* = 0.00^*b*^	4.57 ± 0.50, *Z* = – -7.14, *, P* = 0.00^c^
Spiritual	1.89 ± 0.53	2.03 ± 0.53, *Z* = – 2.32, *P* = 0.00^a^	2.82 ± 0.46, *Z* = – 6.80, *P* = 0.00^b^	3.91 ± 0.58, *Z* = – 7.02, * P* = 0.00^c^
Karnofsky score	73.08 ± 5.57	74.15 ± 6.10, *Z* = – 1.35, *P* = 0.18^a^	77.85 ± 5.15, *Z* = – 4.83, *P* = 0.00^b^	84.31 ± 6.61, *Z* = – 6.48, * P* = 0.00^c^

^a^QoL scores at T2 compared with those at T0; ^b^QoL scores at T6 compared with those at T0; ^c^QoL scores at T10 compared with those at T0. QoL: quality of life

### Tumor response and OS

**Fig. 6:**
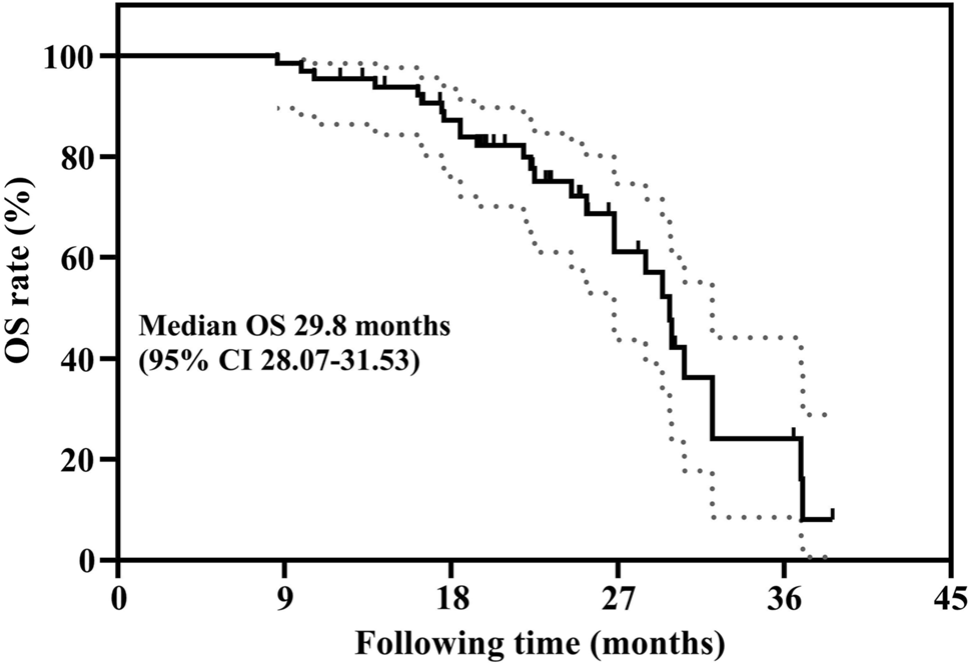
Kaplan–Meier analysis (Log-rank test) was used to analyze the overall survival (OS)

Based on imaging changes at the site of ISI on CT or MRI at T10, among all cases, 2 (3.08%) achieved a CR, 44 (67.69%) achieved a PR, 15 (23.08%) achieved SD, and 4 (6.15%) had PD, which means that the LCR at T10 was 93.85%. The mean postoperative follow-up was 21.90 ± 6.79 months, with a range of 8.60–36.50 months. The median OS was 29.80 ± 0.88 months (95% CI 28.07–31.53). The 1-, 2-, and 3-year OS rates were 95.40%, 75.00%, and 24.10%, respectively (Fig. 6).

## Discussion

According to previous studies, bone pain is the most common type of pain in cancer, which affects approximately 30% of patients with bone metastases [13]. Although there is significant osteosclerosis and increased bone strength in OBMs, the alteration of the bone's internal environment and the destruction of the original mechanical structure changes its mechanical properties, causing more bone fragility [14]. Local structural instability and, thus, compression of peripheral nerves, as well as neurovascular invasion by tumor cells, are the causes of refractory pain in OBMs [15]. EBRT alleviates symptoms and is less toxic, but is slow to relieve pain, resulting in long hospitalization periods and high costs [16]. Moreover, more than 30% of patients do not have pain relief, and nearly 50% had recurrent pain after EBRT as reported by Delgado et al. [17].

Previous studies had shown that ISI was safe and effective in treating cancer pain due to bone metastases, with RoP rates of 65–81% at 3 months [7, 8, 18–20]. In our study, ^125^I brachytherapy significantly relieved cancerous bone pain in patients with OBMs. DMC of all patients was reduced with the extension of irradiation time, which could avoid dose-related toxicities such as nausea and constipation. Similarly, the VAS scores rapidly decreased and the number of patients with severe pain decreased from 47.69% to 15.38% at T2, and the high RoP rates of 84.62% at T6. And a sustained reduction in VAS scores can be observed that the relief of VAS grade to mild pain and the significantly lower analgesic dose in all patients at T10 dose, may be related to the long half-life of ^125^I seeds (59.6 days), which can deliver a continuous dose of 110–160 Gy. This strongly confirmed that effective RoP for OBMs patients with ^125^I brachytherapy in the short term. And, it was consistent with the results of previous studies in which Yao et al. [21] used ^125^I brachytherapy for 82 bone metastases (35 osteolytic, 29 osteoblastic, and 18 mixed), and the VAS scores for the severe pain decreased from 7.0 before treatment to 4.0 at 6 h, and 3.0 at 12 h, fully indicating the RoP efficiency of ^125^I brachytherapy in patients with bone metastases. In another study, DMC for patients at T0 was 175.2 mg, which was decreased to 91.2 mg and 72.8 mg at 2 weeks and 12 weeks, with the efficiency reached 65.3% and 95.2%, respectively [19]. The main mechanisms of ^125^I brachytherapy for cancer-related bone pain may be as follows: (1) killing cancer cells, reducing or terminating the release of pain-causing factors such as bradykinin, prostaglandin, and 5-hydroxytryptamine secreted by tumor cells [22]; (2) cumulative dose irradiation can shrink tumor volume, reduce tumor compression on surrounding nerves and organs and tumor tension; and (3) continuous low-dose radiotherapy causes fibrosis or thrombosis of blood vessels within or adjacent to the tumor and reduces the permeability of blood vessels, thus blocking the penetration of pain-causing factors [20]. Thus, ^125^I brachytherapy is effective for near- to mid-term pain relief in OBMs, but long-term effects or better pain relief in combination with synergistic treatments such as cement augmentation still need further confirmation in prospective studies.

In our study, multivariate logistic regression analysis showed MD and B-ALP were independent influence factors of RoP for OBMs patients with ^125^I brachytherapy. Previous studies have shown that a higher B-ALP was correlated with bone pain in patients with bone metastases at baseline levels [23–25], it can be used as a reference in clinical practice. Moreover, both univariate and multivariate analysis showed that patients with MD < 3 cm could obtain better RoP after receiving ^125^I brachytherapy, suggesting that tumor size was strongly correlated with symptom control. These may be due to the ability of ^125^I seeds to completely kill smaller-sized tumors, relieving their compression of peripheral nerves and the release of pain mediators, and thus providing better symptomatic relief more adequately [19, 22, 27]. This reflects the reality of patients observed in actual clinical practice and may fill a critical gap in knowledge of predictors in patients with smaller tumor sizes (MD < 3 cm) and higher B-ALP (B-ALP ≥ 100 U/L) receiving palliative care.

In OBMs, bone pain has a significant impact on the patient's daily functioning and emotions, resulting in a severe drop in QoL. Patient’s postoperative QoL indicator scores, such as sleep, appetite, fatigue, and spiritual, all improved to varying degrees after receiving ^125^I brachytherapy. However, the Karnofsky scores did not show significant improvements until assessment at T6, which may be attributed to local pain alleviation, a significant decrease in the patient's DMC, and the improvement in the quality of diet and sleep. The marked improvement in the above factors improved the patient's functional status and ultimately influenced their Karnofsky scores.

Although previous studies [26, 28] reported that due to osteosclerosis, OBMs were more difficult to puncture than osteolytic metastases, our center's clinical experience was that an ISI needle was used in conjunction with medical drills for puncturing lesion sites; this greatly reduced the difficulty of puncture, and the technical success rate was 100%. During the operation, CT or CBCT guidance clearly showed the volume of the tumor and the location of vital organs, making the entire operation safer and more accurate. For safety, only minor complications (18.46%) occurred in our study, which was consistent with the previous reports (19%) [9, 19, 21, 27, 29]. This may be due to the efforts applied to ensure the safety of ISI treatment: (1) intraoperative operations under precise guidance of CT or CBCT; (2) the radiation radius of ^125^I seeds is 17 mm, and the radiation dose decays with distance to minimize toxicity to adjacent tissues and organs [28, 30]; and (3) TPS is a key tool for ensuring that this process is carried out without damaging the surrounding tissues, which can meet the requirements of the American Brachytherapy Society’s “dual 90” guideline that a cancer cure requires that 90% of the tumor volume receives a 90% prescription dose [9].

^125^I brachytherapy has also been shown to be effective for tumor control in bone metastases, with a 3-month LCR of 51.7%–100% and a median overall survival of between 10 and 25 months [9, 20, 27, 29–32]. The LCR in the present study was similar to previous studies (93.85%), but the median survival was slightly higher (29.80 months), which is likely due to the difference caused by the heterogeneity of this malignant tissue in contrast to the majority of previous studies that enrolled bone metastases that mostly presented osteolytic changes, whereas the present study was aimed at OBMs. Therefore, the effect of ^125^I brachytherapy on LCR outcome and survival in patients with OBMs needs to be also evaluated by further studies and long-term follow-ups.

However, the study also had some limitations: (1) the nature of this study was retrospective observational and it lacked controls, and prospective research with a larger sample and longer follow-up period was vital; (2) most of the cases were prostate and breast cancers, which were sensitive to brachytherapy, and the heterogeneity of the primary tumor was not analyzed in a stratified manner; and (3) although our findings demonstrated significant improvements in pain control, the follow-up period was short; therefore, further long-term prospective studies are still needed.

## Conclusions

In conclusion, ^125^I brachytherapy is an effective treatment in relieving painful OBMs and improving patients’ QoL. Selection of appropriate tumor size and B-ALP is influential factors in improving the RoP efficacy of ^125^I brachytherapy for OBMs.

## Data Availability

In addition to the raw data in the manuscript, the datasets used are available from the corresponding author on request.

## References

[1] Huang S, Wa QD, Pan JC, Peng XS, Ren D, Li QJ (2018). Transcriptional downregulation of miR-133b by REST promotes prostate cancer metastasis to bone via activating TGF-β signaling. Cell Death Dis.

[2] Kitajima K, Yamamoto S, Fujiwara M, Kawanaka Y, Yamada Y, Nagasawa Y (2021). Accurate monitoring of the response of bone metastases to treatment in patients with prostate cancer using choline PET/CT. Case Rep Oncol.

[3] Wu XQ, Li FF, Dang L, Liang C, Lu AP, Zhang G (2020). RANKL/RANK system-based mechanism for breast cancer bone metastasis and related therapeutic strategies. Front Cell Dev Biol.

[4] Hartsell WF, Scott CB, Bruner DW, Scarantino CW, Ivker RA, Roach M (2005). Randomized trial of short- versus long-course radiotherapy for palliation of painful bone metastases. J Natl Cancer Inst.

[5] Qian JL, Bao ZH, Zou J, Yang HL (2016). Effect of pedicle fixation combined with 125I seed implantation for metastatic thoracolumbar tumors. J Pain Res.

[6] Jiang AG, Lu HY, Ding ZQ (2019). Implantation of 125I radioactive seeds via c-TBNA combined with chemotherapy in an advanced non-small-cell lung carcinoma patient. BMC Pulm Med.

[7] Shi F, Li W, Zhang X, Rakesh M, Li CX, Zhang FJ (2015). 125I seed implant brachytherapy for painful bone metastases after failure of external beam radiation therapy. Medicine (Baltimore).

[8] Xiang ZW, Wang LF, Yan HZ, Zhong ZH, Liu WK, Mo ZQ (2018). 125I seed brachytherapy versus external beam radiation therapy for the palliation of painful bone metastases of lung cancer after one cycle of chemotherapy progression. Onco Targets Ther.

[9] Liu Y, He C, Li Y, Chen YX, Yang L, Li TY (2019). Clinical efficacy of computed tomography-guided iodine-125 seed implantation therapy for patients with metastatic epidural spinal cord compression: a retrospective study. J Cancer Res Ther.

[10] Swarm RA, Paice JA, Anghelescu DL, Are M, Bruce JY, Buga S (2019). Adult cancer pain, version 3.2019, NCCN clinical practice guidelines in oncology. J Natl Compr Canc Netw..

[11] Seyal AR, Gonzalez-Guindalini FD, Arslanoglu A (2015). Reproducibility of mRECIST in assessing response to transarterial radioembolization therapy in hepatocellular carcinoma. Hepatology.

[12] CIRSE 2020 Summit–Book of Abstracts. Cardiovasc Intervent Radio. 2020;43(Suppl 4):127–459. 10.1007/s00270-020-02606-2 10.1007/s00270-020-02606-2 32892242

[13] Xu H, Peng C, Chen XT, Yao YY, Chen LP, Yin Q (2020). Chemokine receptor CXCR4 activates the RhoA/ROCK2 pathway in spinal neurons that induces bone cancer pain. Mol Pain.

[14] Kane CM, Hoskin P, Bennett MI (2015). Cancer induced bone pain. BMJ.

[15] Gao QP, Yang DZ, Yuan ZB, Guo YX (2021). Prognostic factors and its predictive value in patients with metastatic spinal cancer. World J Clin Cases.

[16] Zhao GS, Liu S, Yang L, Li C, Wang RY, Zhou J (2020). Evaluation of radioactive 125I seed implantation for the treatment of refractory malignant tumors based on a CT-guided 3D template-assisted technique: efficacy and safety. BMC Cancer.

[17] Delgado-López PD, Roldán-Delgado H, Corrales-García EM (2020) Stereotactic body radiation therapy and minimally invasive surgery in the management of spinal metastases: a change in the paradigm. Neurocirugia (Astur: Engl Ed). 31(3):119–131. 10.1016/j.neucir.2019.08.005 10.1016/j.neucir.2019.08.005 31668627

[18] Jiao DC, Wu G, Ren JZ, Han XW (2016). Radiofrequency ablation versus 125I-seed brachytherapy for painful metastases involving the bone. Oncotarget.

[19] Wang W, Liu ZH, Zhu JR, Wu CW, Liu MY, Wang YZ (2017). Brachytherapy with iodine 125 seeds for bone metastases. J Cancer Res Ther.

[20] Xiang ZW, Mo ZQ, Li GH, Gilani S, Zhong ZH, Zhang T (2016). 125I brachytherapy in the palliation of painful bone metastases from lung cancer after failure or rejection of conventional treatments. Oncotarget.

[21] Yao Y, Li ZM, Jiao DC, Zhou XL, Li J, Han XW (2021). Palliative local treatment of bone metastases by 125I seed brachytherapy under DynaCT guidance: single-center experience. Diagn Interv Radiol.

[22] Xie L, Chen YJ, Zhang Y, Yang ZZ, Zhang ZX, Shen LD (2015). Status and prospects of percutaneous vertebroplasty combined with 125I seed implantation for the treatment of spinal metastases. World J Surg Oncol.

[23] Kuchuk I, Beaumont JL, Clemons M, Amir E, Addison CL, Cella D (2013) Effects of de-escalated bisphosphonate therapy on the functional assessment of cancer therapy-bone pain, brief pain inventory and bone biomarkers. J Bone Oncol 8;2(4):154–157. 10.1016/j.jbo.2013.07.004 10.1016/j.jbo.2013.07.004 PMC4723387 26909286

[24] Froberg MK, Garg UC, Stroncek DF, Geis M, McCullough J, Brown DM (1999). Changes in serum osteocalcin and bone-specific alkaline phosphatase are associated with bone pain in donors receiving granulocyte-colony-stimulating factor for peripheral blood stem and progenitor cell collection. Transfusion.

[25] Karhade AV, Thio QCBS, Kuverji M, Ogink PT, Ferrone ML, Schwab JH (2019). Prognostic value of serum alkaline phosphatase in spinal metastatic disease. Br J Cancer.

[26] He J, Mai Q, Yang F, Zhuang W, Gou Q, Zhou Z (2021). Feasibility and clinical value of CT-guided 125I brachytherapy for pain palliation in patients with breast cancer and bone metastases after external beam radiotherapy failure. Front Oncol.

[27] Zhang L, Lu J, Wang Z, Cheng Y, Teng G, Chen K (2016). Clinical efficacy of computed tomography-guided iodine-125 seed implantation therapy in patients with advanced spinal metastatic tumors. OncoTargets Ther.

[28] Sharma R, Sagoo NS, Haider AS, Sharma N, Haider M, Sharma IK (2021). Iodine-125 radioactive seed brachytherapy as a treatment for spine and bone metastases: A systematic review and meta-analysis. Surg Oncol.

[29] Yang ZR, Chen G, Cui Y, Su TH, Yu JN, Xiao GW (2019). Iodine-125 seed implantation combined with arterial chemoembolization therapy for pain palliation in metastatic bone cancer: a retrospective study. Cancer Biol Ther.

[30] Lu J, Huang W, Wang Z, Gong J, Ding X, Chen Z (2018). The safety and efficacy of interstitial 125I seed implantation brachytherapy for metastatic epidural spinal cord compression. J Cancer Res Ther.

[31] Yao L, Cao Q, Wang J, Yang J, Meng N, Guo F (2016). CT-guided 125I seed interstitial brachytherapy as a salvage treatment for recurrent spinal metastases after external beam radiotherapy. Biomed Res Int.

[32] Yang Z, Tan J, Zhao R, Wang J, Sun H, Wang X (2013). Clinical investigations on the spinal osteoblastic metastasis treated by combination of percutaneous vertebroplasty and (125)I seeds implantation versus radiotherapy. Cancer Biother Radiopharm.

